# Progress in Surgery

**Published:** 1896-03-28

**Authors:** 


### Progress in Surgery.

### SURGERY OF THE VERMIFORM APPENDIX.

**Anatomy.**—Berry,[1] from investigating 100 cases, finds the length of the appendix to average 8·3 cm., but the average obtained from all observations is 9·2 cm. As regards the relation of the length to sex, he supports Bryant's statement that the appendix is longer in the male. The greatest length is obtained between 20 and 40 years of age. The diameter is a point of considerable physiological and pathological importance, and by external measurement he finds it is about 6 mm. at the base and centre, and 5 mm. at the apex. It is about 1 mm. wider in the male than in the female. Berry asserts the correctness of the generally-accepted statement that the cavity of the appendix is obliterated at, or shortly after, middle age; and believes that the obliteration is a physiological process. He advances in support of his opinion the fact that a large part of the appendix consists of lymphoid tissue, which is known to undergo atrophy after a certain stage of life.

**Appendicitis.**—Honel[2] says the chief indications for operation are the presence of pus, peritonitis from perforation, and recurrent attacks. The presence of pus is not always easy of recognition. Neither the pulse nor the temperature, nor the local swelling, presents in every case diagnostic evidence. The author draws a comparison between the medical and surgical treatment of the affection, and while it is admitted that the great majority of the cases recover from the attack, it is stated that many of these suffer recurrence of the disease. Dawbarn[3] thus describes his method of dealing with the stump after removal of the appendix: (1) A continuous Lembert's suture of silk is made to surround the appendix, running like a purse-string or gathering-string in the superficial layers of the cæcum, one-fourth of an inch from the appendix. This suture is not yet tightened, although the first half of a surgeon's knot is already made. (2) The appendix is divided, leaving a stump of variable length, but never shorter than a half-inch. (3) This stump is stretched for a moment gently by introducing through its calibre a closed pair of slender, mouse-toothed forceps, passing them into the cæcum and gently opening the blades; thereby any stricture from swelling of mucous membrane or from plastic deposit will be stretched, and the next step will be easier in consequence. The stump is seized at the extremity of its free end by a similar fine-pointed pair of mouse-toothed forceps, and the stump is promptly invaginated—turned "outside in"—as a glove finger might be, so that when completed the forceps and appendix-end are one-half inch inside the cæcum. (5) The suture is now tightened, during which step the forceps are withdrawn. Sometimes it is a help to insert a probe or grooved director between the open jaws of the forceps prior to withdrawing the latter, in order to prevent the appendix drawing itself out again with them. Two points in the technique should be noted: the use of dry sterile gauze to hold the viscera, to prevent slipping, and pressure of the thumb and finger upon the cæcum near the appendicular attachment to prevent the escape of fæcal contents during the manipulations. Edebohls[4] practically adopts the same method, but in some cases invaginates the whole of the appendix and not merely the stump. He submits the following propositions: (1) Inversion of the stump of the appendix should be substituted for ligation in all cases of acute appendicitis in which it can be applied. (2) In chronic appendicitis either the entire appendix or its stump should be inverted, preference being given to inversion of the uncut appendix whenever practicable. (3) In all cœliotomies undertaken for the relief of conditions other than appendicitis, the normal appendix, if readily and safely accessible, should be inverted entire. Caley[5] thinks that apart from cases which may be accurately termed "relapsing," the liability to recurrence in appendix disease renders it imperative that an individual who has once had an attack of appendicitis should be instructed as to the paramount necessity for strict attention to such matters as are calculated to render recurrence less probable, *e.g.*: (1) The digestion must be attended to, the meals must be taken at regular hours and not hurried, the food must be simple and digestible, and of such a kind as to leave as little *débris* as possible in the intestine. (2) The bowels must be made to act daily. The aperient given should be frequently changed. (3) Massage of the abdomen appears to have in many cases a very admirable effect. This may be associated with suitable exercise. (4) The use of some intestinal antiseptic. Salol in 10-grain doses night and morning (in milk or in a cachet) appears the most efficient. Reynier[6] thinks that when an operation is performed it should be restricted to evacuation of the pus, so as not unnecessarily to add to the danger of the original disease by complicated manœuvres. With regard to the opportune moment for operation, he thinks that an immediate operation increases the risk of generalised peritonitis, whereas, if the intervention is delayed two or three days, adhesions have time to form, while the interval may be profitably employed in improving the patient's strength. Reclus[7] and Poirier[8] agree in thinking that the best incision is that recommended by Roux, as close to Poupart's ligament as possible. The former advises always to endeavour to find the appendix, not for the purpose of removing it, but in order to be able to open any abscess which may exist in the neighbourhood. Murray[9] gives an account of twenty-three cases in children, chiefly interesting, because their subsequent history is known, and because, with one exception, no case is more recent than two years ago. There were nine patients in whom there was inflammation of the vermiform appendix, with most probably a slight and strictly localised peritonitis; these all recovered under medical treatment, and remain well, though three of them were relapsing cases. The period that has elapsed since their admission varies from three to seven years. Ten cases where there was a localised peritoneal abscess due to inflammation of the appendix, the abscess was simply opened and drained. Nine of them remain well, the time that has elapsed since operation varying from three to six years. One died from suppurative peritonitis owing to an accident during the syringing of the wound. Three cases where the appendix rapidly became gangrenous or perforated; all of them died. Mundé[10] thinks that the numerous instances of appendicitis, with and without suppuration, occurring during pregnancy and labour, which have been reported, should induce us to watch for this accident at those times quite as much as on other occasions, and to treat the disease entirely regardless of the existence of pregnancy. He does not agree with those surgeons who would induce abortion or premature labour during, and on account of, appendicitis. Cathcart[11] has formed the following conclusions: (1) The lumen of the appendix is apt to become narrowed or blocked at some point either with or without the presence of a calculus or concretion. (2) The blockage may exist without causing symptoms, but may at any moment give trouble, without evident exciting cause, and without warning. (3) The dangerous factor in such cases is not so much mere accumulation of fluid behind the obstruction as the fermentation of the fluid, for he has found inflammation of the coats of the appendix in the obstructed part, and beyond it, ulceration of the mucous membrane behind the obstruction, as well as impending gangrene and perforation, and such changes would not result from mere distension. (4) Peritonitis may occur without perforation. (5) After a severe onset a lull in the severity of the symptoms may be consistent with steady progress of the inflamed appendix towards gangrene. Tuffier[12] regards digital exploration of the rectum as of great importance in appendicitis, as by this means, combined with abdominal palpation, he has on several occasions discovered the existence in the true pelvis of a large mass, which could not be detected simply by palpation of the abdomen. Quénu,[13] on the other hand, is strongly opposed to digital exploration of the rectum, because as a rule the surgeon is obliged to perform an operation at the same sitting, and infection resulting from this exploration may have very serious consequences. The method of operation which Quénu employs consists in incision of the abdominal wall down to the peritoneum, opening the abscess, if there are adhesions; if, on the contrary, no adhesions exist, a gauze pledget is merely left in contact with the purulent focus. By this method he has found that the abscess has never once opened except into the wound.

[1] Med. Chron., Dec., 1895; and Anatom. Anzeig., No. 24, 1895. [2] Amer. Jour., Med. Sci., Oct., 1895; and Munich. Med. Woch., 1895, No. 13 and 14. [3] Amer. Jour. Med Sci., Aug., 1895; and Internat. Jour. of Surg., 1895, vol. viii, No. 8. [4] Amer. Jour. Med. Sci., June, 1895. [5] Practitioner, Aug., 1895, [6] Med. Week, Aug 9, 1895. [7] Ibidem. [8] Ibidem. [9] Brit. Med. Jour., Nov. 9, 1895. [10] New York Med. Rec., Oct. 26, 1895. [11] Edin. Med. Jour., Nov., 1895. [12] Med. Week, Oct. 25, 1895. [13] Ibidem.

